# Sequential treatment with a TNFR2 agonist and a TNFR1 antagonist improves outcomes in a humanized mouse model for MS

**DOI:** 10.1186/s12974-023-02785-y

**Published:** 2023-05-03

**Authors:** Valentina Pegoretti, Jan Bauer, Roman Fischer, Iskra Paro, Wanda Douwenga, Roland E. Kontermann, Klaus Pfizenmaier, Evelien Houben, Bieke Broux, Niels Hellings, Wia Baron, Jon D. Laman, Ulrich L. M. Eisel

**Affiliations:** 1 Department of Molecular Neurobiology, Groningen Institute of Evolutionary Life Science (GELIFES), University of Groningen, 9747 AG Groningen, The Netherlands; 2 Institute of Cell Biology and Immunology, University of Stuttgart, 70569 Stuttgart, Germany; 3 Division of Neuroimmunology, Center for Brain Research, Medical University of Vienna, 1090 Vienna, Austria; 4 Neuroimmune Connections and Repair (NIC&R) Lab, Department of Immunology and Infection, Biomedical Research Institute, Hasselt University, 3590 Hasselt, Belgium; 5 University MS Centre, 3590 Hasselt/Pelt, Belgium; 6 Department Biomedical Sciences of Cells and Systems (BSCS), Section Molecular Neurobiology, University Medical Center Groningen, 9713 GZ Groningen, The Netherlands; 7 Department Pathology and Medical Biology, University Medical Centre Groningen (UMCG), University of Groningen, 9713 GZ Groningen, The Netherlands; 8 Stuttgart Research Centre Systems Biology, University of Stuttgart, 70569 Stuttgart, Germany

**Keywords:** TNF, TNFR1 antagonist, TNFR2 agonist, MS, EAE, Neuroinflammation

## Abstract

TNF signaling is an essential regulator of cellular homeostasis. Through its two receptors TNFR1 and TNFR2, soluble versus membrane-bound TNF enable cell death or survival in a variety of cell types. TNF-TNFRs signaling orchestrates important biological functions such as inflammation, neuronal activity as well as tissue de- and regeneration. TNF-TNFRs signaling is a therapeutic target for neurodegenerative diseases such as multiple sclerosis (MS) and Alzheimer’s disease (AD), but animal and clinical studies yielded conflicting findings. Here, we ask whether a sequential modulation of TNFR1 and TNFR2 signaling is beneficial in experimental autoimmune encephalomyelitis (EAE), an experimental mouse model that recapitulates inflammatory and demyelinating aspects of MS. To this end, human TNFR1 antagonist and TNFR2 agonist were administered peripherally at different stages of disease development in TNFR-humanized mice. We found that stimulating TNFR2 before onset of symptoms leads to improved response to anti-TNFR1 therapeutic treatment. This sequential treatment was more effective in decreasing paralysis symptoms and demyelination, when compared to single treatments. Interestingly, the frequency of the different immune cell subsets is unaffected by TNFR modulation. Nevertheless, treatment with only a TNFR1 antagonist increases T-cell infiltration in the central nervous system (CNS) and B-cell cuffing at the perivascular sites, whereas a TNFR2 agonist promotes Treg CNS accumulation. Our findings highlight the complicated nature of TNF signaling which requires a timely balance of selective activation and inhibition of TNFRs in order to exert therapeutic effects in the context of CNS autoimmunity.

**Supplementary Information:**

The online version contains supplementary material available at 10.1186/s12974-023-02785-y.

## Introduction

Tumor necrosis factor-alpha (TNF) is a master cytokine involved in many, sometimes opposing, cellular signaling pathways such as apoptosis, cell survival, inflammation and tissue regeneration [1–5]. Currently, anti-TNF therapies are widely used as FDA-approved treatments for several diseases such as Crohn’s disease (CD), ulcerative colitis (UC), rheumatoid arthritis (RA), ankylosing spondylitis (AS), psoriatic arthritis, and plaque psoriasis [6]. The efficacy of these treatments varies in different diseases. Further, patients can become unresponsive, and can experience adverse reactions, such as infections and injection reactions. Moreover, a phase II clinical study testing a recombinant TNF receptor 1 (TNFR1) immunoglobulin fusion protein neutralizing TNF (lenercept) in patients with multiple sclerosis (MS) had to be halted due to exacerbation of symptoms when compared to placebo-treated MS patients [7]. Hence, although partially effective in various autoimmune diseases, anti-TNF therapies in MS patients seem to worsen pathology and clinical symptoms. MS seems to require therapeutics that diminish the inflammatory response but also directly promote tissue protection and regeneration.

A possible explanation for the failure of anti-TNF therapies in MS is the ability to neutralize TNF regardless of its various downstream functions. There are two molecular variants of TNF, namely a transmembrane-bound TNF (tmTNF) and a soluble TNF (sTNF), displaying different affinities for the two receptors: tmTNF is the preferable ligand of TNFR2 [8] and sTNF exhibits higher affinity for TNFR1 [9]. TNFRs are involved in many physiological functions [1, 10–12] and are differentially expressed on the surface of many different cell types such as immune [13–15] and central nervous system (CNS) cells [16–18]. Furthermore, the two receptors differ in the intracellular signaling pathways they trigger, thereby leading to different cellular responses. In general, tmTNF stimulates cell survival and proliferation through TNFR2 activation [19], whereas sTNF initiates apoptotic and pro-inflammatory signals via TNFR1 [20].

Accordingly, several in vivo studies highlight the potential of targeting TNFRs selectively to restore homeostasis in different animal models for neurodegenerative and inflammatory diseases, including MS [1, 10, 21–26].

In a cuprizone model for de- and re-myelination, specific TNFR knock-out (KO) mice reveal opposing functions of TNFRs: TNFR1 KO reduces demyelination and axonal damage, whereas TNFR2 KO decreases remyelination [27]. Furthermore, evidence from transgenic mice highlighted a protective role of TNFR2 in oligodendrocytes proliferation and differentiation [27, 28] while TNFR1 has a detrimental role promoting demyelination and inflammation [5, 12, 29]. In particular, the experimental autoimmune encephalomyelitis (EAE) mouse model recapitulates aspects of MS pathology such as autoimmune response against a myelin antigen, immune-mediated tissue injury and CNS inflammation [30, 31]. In the EAE model, selective antibody-mediated TNFR1 inhibition on the day of immunization attenuates symptoms severity and delays disease onset, due to decreased demyelination and neuronal loss [32]. Likewise, three injections of a TNFR2 agonist before symptoms onset in this paradigm accelerate remyelination and greatly diminish motor and sensory deficits through reduction of central and peripheral inflammation [23]. Therefore, the role of TNF-TNFRs signaling in autoimmune and neurodegenerative diseases such as MS has been demonstrated [1, 22, 33–35].

To assess the efficacy of human-specific compounds in disease mouse models, chimeric human/murine TNFR knock-in (hu/m TNFR ki) mouse lines expressing the extracellular part of TNFRs human and intracellular part of mouse TNFRs were engineered (B6.B6-huTNFRSF1A_ecd_^tm1UEG^ and B6.B6huTNFRSF1B_ecd_^tm2UEG^) [22]. Using these humanized mice, we previously showed that a human TNFR2-selective agonist (EHD2-scTNF_R2_) and a human TNFR1-selective antagonistic antibody (ATROSAB) reduced neuroinflammation and neuronal loss in a NMDA-induced nucleus basalis lesion model [22]. Furthermore, ATROSAB improved clinical symptoms of humanized TNFR1 EAE mice and decreased leukocyte CNS infiltration, demyelination and axonal damage [36]. Hence, selective targeting of TNF-TNFRs signaling holds great promise for treatment of MS as well as other inflammatory and neurodegenerative diseases.

The aim of this study is to investigate whether modulation of both TNFRs in humanized TNFRs knock-in mice outperforms single treatments in impeding EAE development. We demonstrate that early treatment with a human TNFR2 agonist (EHD2-scTNF_R2_) enhances the therapeutic effects of a human TNFR1 antagonist (ATROSIMAB) on EAE symptoms. The sequential strategy decreases demyelination in these animals while peripheral immune cell subsets are unaffected. Of interest, we observe increased numbers of T cells and B-cell cuffs in the spinal cord upon single ATROSIMAB treatment. This effect is reversed when animals are co-treated with EHD2-scTNF_R2_, suggesting a role of TNFRs in regulating lymphocyte recruitment to the CNS in an autoimmunity disease context. This study reports new findings on the modulation of TNF-TNFRs signaling in a mouse model for MS and highlights the potential but also the challenges of this therapeutic strategy for MS patients.

## Materials and methods

### Materials

The human TNFR2 agonist EHD2-scTNF_R2_ and the human TNFR1 antagonist ATROSIMAB were produced as previously described [22, 37]. The mouse monoclonal immunoglobulin (Ig) G1 antibody (HM1097) against TNFR1 was purchased from Hycult Biotech (Uden, The Netherlands). Details regarding the primary antibodies used for immunohistochemistry and flow cytometry are summarized in Additional file 1: Table S1. Luxol Fast Blue (LFB) staining was used to determine the degree of demyelination. Primers for g**e**notyping used to distinguish human and mouse TNFR1 (5′-CTAAACATTCCTTGACCGGC-3′; 5′-TTCCCACACAAATCTTGACG-3′; 5′-ATGCTAGGGACAACAGCCAG-3′) and TNFR2 (5′-GGTCCAAACCTTCTAAGCCC-3′; 5′-ACATCAATATAGGCCAGCCG-3′; 5′-GCGTAGGGTGTAAATGCCAC-3′) were obtained from Life Technologies (Carlsbad, USA).

### Animals

Human/murine TNFR1 knock-in (B6.B6-huTNFRSF1A_ecd_^tm1UEG^, hu/m TNFR1-ki) and human/murine TNFR2 knock-in (B6.B6-huTNFRSF1B_ecd_^tm2UEG^, hu/m TNFR2-ki) mice were generated by Ozgene Pty Ltd (Bentley, Australia) as previously described [22]. For the double ki mouse line, homozygous hu/m TNFR1-ki mice were crossed with homozygous hu/m TNFR2-ki mice for at least 10 generations to obtain hu/m TNFR1-ki x hu/m TNFR2-ki mice. In these transgenic mouse lines, the mouse sequence coding for the extracellular part of the TNFR is replaced with the human one while the sequence coding for the intracellular part is mouse. Both endogenous mouse and exogenous human TNF are able to bind and activate the human part of the receptor, thereby triggering the typical murine intracellular signaling pathways. Transgenic mouse lines as well as C57BL/6 (WT) mice were bred and housed in the same room at the FSE animal facility. Animals were group-housed on a 12-h light/12-h dark schedule and had food and water available ad libitum. Animal experiments were carried out in accordance with the European Directive (2010/63/EU) on the protection of animals used for scientific purposes.

### Experimental autoimmune encephalomyelitis (EAE) and treatment regimen

**Fig. 1:**
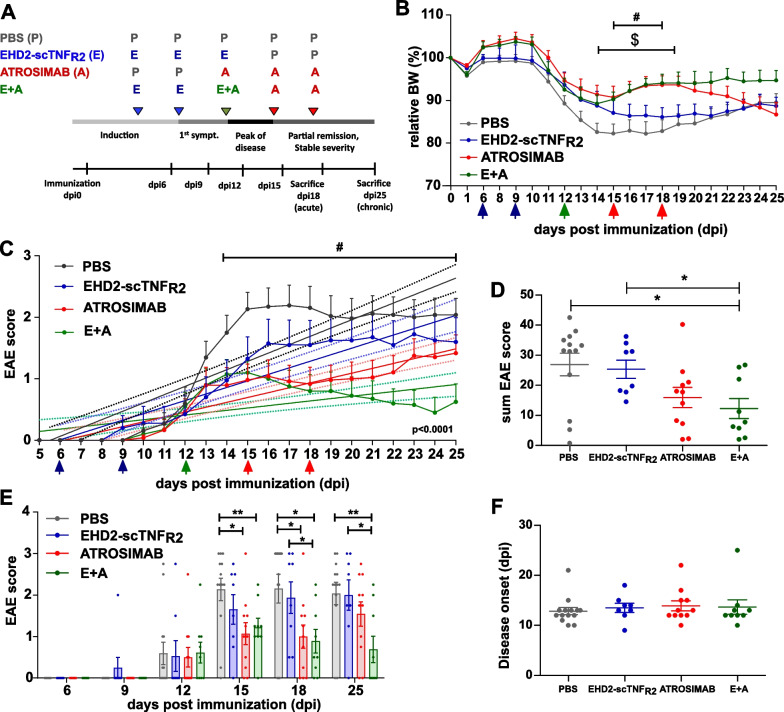
EHD2-scTNF_R2_ enhances the therapeutic effect of ATROSIMAB on EAE. Hu/m TNFR1-ki x hu/m TNFR2-ki mice were immunized with MOG_35-55_ and treated either with saline (*n* = 13), EHD2-scTNF_R2_ (TNFR2 agonist; *n* = 8), ATROSIMAB (TNFR1 antagonist; *n* = 11) or a combination (E + A; *n* = 9). Schematic representation of EAE induction, development and treatment regimen (**A**). EHD2-scTNF_R2_ [10 mg/kg] was injected i.p. at 6, 9 and 12 days post-immunization (dpi; blue and green arrows) while ATROSIMAB [20 mg/kg] was injected at 12, 15 and 18 dpi (red and green arrows). Body weight (BW; **B**) and disease development (**C**) were measured daily until 25 dpi. EAE development is represented as sum of EAE scores over 25 days (**D**) but also as mean score at the different treatment days and at killing day (**E**). On the first day of symptoms, disease onset was recorded (**F**). Linear regression curves of disease development (**C**) are shown together with dashed lines representing 95% confidence intervals. Statistical difference between slopes is shown at the bottom right of the graph. Data are presented as mean ± SEM and differences between groups were assessed with Mann–Whitney or Kruskal–Wallis tests, except for relative body weight for which two-way ANOVA and Tukey’s post-test were used. # = PBS vs E + A; $ = PBS vs ATROSIMAB. *^/#/$^*p* < 0.05, **^/##/$$^*p* < 0.01

Female mice (10–12 weeks old) were anesthetized by inhalation of 4% isoflurane and immunized with MOG_35–55_ peptide emulsified in complete Freund’s adjuvant (Hooke Labs Inc; #EK-2110) with one subcutaneous (s.c.) injection at the upper and one at the lower back. Subsequently, the immunized mice received intraperitoneal (i.p.) injection with pertussis toxin (PTX) at 2 h and 24 h after immunization. To asses ascending paralysis, clinical symptoms were scored daily for either 18 or 25 days on a scale from 0 to 5 (0: no obvious changes, 1: limp tail, normal movement, 2: falls through the grate, no grabbing and wobbling when walking, 3: hind legs are partially paralyzed, 4: hind legs completely paralyzed, 5: front legs are also completely paralyzed). Reasons for exclusion from the studies included sudden death due to severe EAE symptoms, humane end point, no disease development, death caused by anaphylaxis after 3rd treatment injection and disease onset before first treatment injection. Hu/m TNFR2-ki mice received i.p. injections 9 and 13 dpi with either PBS, human EHD2-scTNF_R2_ [10 mg/kg], mouse anti-TNFR1 [20 mg/kg] or a combination of the last two compounds. Control WT mice received PBS injections at 9 and 13 dpi. Hu/m TNFR1-ki x hu/m TNFR2-ki were treated i.p. with PBS, EHD2-scTNF_R2_ [10 mg/kg], ATROSIMAB [20 mg/kg] or a combination. EHD2-scTNF_R2_ and ATROSIMAB treatments were injected at 6, 9, 12 or 12, 15, 18 dpi, respectively. PBS was injected on the other treatment days (Fig. 1A). Clinical EAE scoring was performed by two investigators blinded to treatment. The EAE cumulative score per animal was calculated as area under the curve (AUC).

### Histopathology and immunohistochemistry

After i.p. injection of 20% sodium pentobarbital, mice were transcardially perfused with 0.5% heparin physiological saline followed by 4% paraformaldehyde (PFA) in PBS solution. Brains and spinal cords were removed, postfixed for 24 h with 4% PFA in PBS, washed three times with PBS and subsequently stored in PBS at 4 °C for at least 24 h. Immunohistochemical staining was performed on paraffin embedded tissue. Paraffin sections were cut into 3–5 µm, deparaffinized and stained with Luxol Fast Blue/Periodic Acid Schiff to assess demyelination. Demyelination in the spinal cord white matter was measured on a total of 5–10 cross sections by using a 10 × 10 morphometric grid at 5 × magnification and counting the myelinated and demyelinated areas. Finally, demyelination is presented as a percentage of total white matter. For immunohistochemical staining, 3- to 5-µm-thick paraffin sections were deparaffinized in xylene and transferred to 90% ethanol. Endogenous peroxidase was blocked by 30-min incubation in methanol with 0.02% H_2_O_2_. Sections were then transferred to distilled water via a 90%, 70% and 50% ethanol series. Before staining with antibodies, antigen retrieval was performed by heating the sections for 60 min in a plastic Coplin jar filled with EDTA (0.05 M) in TRIS buffer (0.01 M, pH 8.5) in a household food steamer device (MultiGourmet FS 20, Braun, Kronberg im Taunus, Germany). After antigen retrieval, sections were incubated with 10% fetal calf serum (FCS) in wash buffer (Agilent Dako Omnis, Santa Clara, USA). Next, primary antibodies were applied in FCS/wash buffer at 4 °C overnight. Immunoglobulin was detected by staining with biotinylated anti-mouse IgG (Jackson ImmunoResearch Laboratories Inc., West Grove, USA). After washing with PBS, secondary antibodies in FCS/wash buffer were applied for 1 h at room temperature. We used biotinylated donkey anti-rabbit, anti-rat or anti-mouse secondary antibodies at a concentration of 1:500 (Jackson ImmunoResearch Laboratories Inc., West Grove, USA). As a third step, avidin peroxidase (1:100; Sigma-Aldrich, St. Louis, USA-Aldrich, St. Louis, USA) was used. Labeling was visualized with 3,3′ diaminobenzidine-tetra-hydrochloride (DAB; Sigma-Aldrich, St. Louis, USA-Aldrich, St. Louis, USA).

Image analysis was performed blinded and using the open-source software QuPath (https://qupath.github.io) either by automated counting of CD3 and FoxP3-positive cells (positive cell detection plugin) or by manually counting B-cell cuffs.

### Isolation of primary immune cells from spleen and lymph nodes

Spleen and iLN were collected before PFA perfusion in RPMI 1640 (Lonza, Basel, Switzerland) supplemented with 1% penicillin–streptomycin (PS, Sigma-Aldrich, St. Louis, USA). While iLN were isolated after saline perfusion, spleens were collected after ligating the splenic artery before fixation. Spleens and iLN were dissociated through a 70-µm filter. After centrifugation at 300×*g*, dissociated cells from iLN were resuspended in RPMI 1640 with 1% PS. Splenocytes were centrifuged at 300×*g* and resuspended in 0.83% ammonium chloride dissolved in double-distilled water. After an incubation time of 4 min, splenocytes were washed with RPMI 1640 plus 1% PS and passed again through a 70-µm filter. Isolated cells from both organs were counted before plating at a density of one million cells per well (U-bottom 96-well plate). Before staining, cells were stimulated in vitro to produce cytokines with 20 ng/ml of PMA (Sigma-Aldrich, St. Louis, USA), 1 µg/ml of ionomycin (CaI, Sigma-Aldrich, St. Louis, USA) and 2 µg/ml Golgiplug (BD Biosciences, Franklin Lakes, USA) for 4 h at 37 °C. Thus, cells were separated and counted with flow cytometry based on the cytokines they can produce once activated.

### Flow cytometry

Live cells were separated from dead cells with Zombie NIR (Biolegend, San Diego, USA) staining. Next, cells were washed with PBS and incubated with 10% rat serum in PBS for 15 min. Extracellular staining was performed using fluorescently labeled antibodies listed in Additional file 1: Table S1 in FACS buffer (PBS supplemented with 1% fetal bovine serum and 0.5% sodium azide 20%). For intracellular staining, cells were fixed using Cytofix/Cytoperm™ kit (BD Biosciences, Franklin Lakes, USA) for 30 min at 4 °C. Next, cells were firstly washed with Perm/Wash™ buffer (P/W, Biolegend, San Diego, USA) and then incubated with the proper intracellular stains (Additional file 1: Table S1) diluted in P/W buffer. After two steps of washing with P/W buffer, cells were resuspended in FACS buffer and measured using BD FACSDiva™ (BD Biosciences, Franklin Lakes, USA). Raw data were analyzed using FlowJo™ v10.8 Software (BD Life Sciences, Franklin Lakes, USA) and Additional file 1: Fig. S1 shows the gating strategy used.

### ELISA for the quantification of anti-drug antibodies in serum

Blood was withdrawn before immunization from the tail vein and before killing via cardiac puncture. Plasma samples were stored at − 80 °C after a 2000×*g* centrifugation step to remove red and white blood cells. High binding ELISA plates (Greiner, Kremsmünster, Austria) were coated with either ATROSIMAB (3 µg/ml) or EHD2-scTNF_R2_ (3 µg/ml) diluted in PBS and incubated overnight in the fridge. After four washing steps, residual binding sites were blocked for 2 h with 2% skim milk in PBS with 0.05% Tween-20 followed by incubation for 2 h with serum samples diluted 1:512 in the same blocking buffer. Samples were then incubated with HRP-conjugated anti-mouse IgG [Fc-specific] (A2554, lot. #045M4780V, Sigma-Aldrich, St. Louis, USA) diluted 1:10.000 in blocking buffer. Binding was detected by using 3, 3′, 5, 5′ tetramethyl benzidine (TMB substrate set, Biolegend, San Diego, USA). The enzymatic reaction was stopped with 1 M sulfuric acid and absorbance at 450 nm was determined. Between each step, plates were washed three times with 0.05% Tween-20 in PBS.

### Statistics

Data are presented as mean ± standard error of the mean (SEM). Normal distribution was assessed by Shapiro–Wilk normality test. Statistical analyses were performed by Mann–Whitney test, Kruskal–Wallis test or one-way ANOVA, followed by a post hoc Tukey’s multiple comparisons post hoc test to compare two groups when data were not normally distributed or variances were unequal. Differences between treatments on EAE development over time were assessed using simple linear regression analysis. Two-way ANOVA was performed to measure differences in body weight loss between all groups at different timepoints. A value of *p* < 0.05 was considered statistically significant. Graphs were plotted using GraphPad Prism Software v8 (San Diego, California USA).

## Results

### TNFR2 activation followed by TNFR1 inhibition decreases EAE symptoms and body weight loss more effectively than single treatments

Previous data show that blocking TNFR1 has a therapeutic effect on EAE symptoms and pathology [21, 36]. Likewise, stimulating TNFR2 promotes recovery from EAE-induced motor and sensory impairments [23]. Therefore, we investigated whether modulating both TNFRs has a stronger effect than single treatments in impeding EAE development using humanized TNFR ki mice. As previously shown in humanized TNFR1 ki mice [36], humanized TNFR2 ki mice develop EAE similarly to WT mice but they tend to lose more weight during disease development (Additional file 1: Fig. S2A–D). When mice are co-treated with a mouse anti-TNFR1 and a human TNFR2 agonist (EHD2-scTNF_R2_), symptoms and demyelination decrease to a similar extent as in mice treated with only TNFR1 antagonist, at least until 18 days post-immunization (dpi; Additional file 1: Fig. S2 A, B and F). Both anti-TNFR1 and co-treatment groups show a higher number of CD3 + T cells in the spinal cord (SC; Additional file 1: Fig. S2 H) compared to saline group, while blocking TNFR1 promotes B-cell cuffing at perivascular sites (Additional file 1: Fig. S2 J).

Since stimulating TNFR2 decreases motor symptoms most effectively when given before rather than after symptoms onset [23], we next asked whether TNFR2 agonist treatment should precede anti-TNFR1 treatment in order to enhance therapeutic effects on EAE symptoms and pathology.

In this study, we sequentially treated humanized TNFR1 and TNFR2 ki mice with human compounds (Fig. 1A): ATROSIMAB is a monovalent antagonistic antibody that selectively blocks TNFR1 signaling [37] while EHD2-scTNF_R2_ is a fusion protein resembling tmTNF and thus, specifically activates TNFR2 [22]. Early stimulation of TNFR2 enhances the therapeutic effects of blocking TNFR1 on EAE symptoms and body weight loss (Additional file 1: Table S2, Fig. 1B–D). While three injections of EHD2-scTNF_R2_ slightly decrease EAE symptoms, ATROSIMAB exerts therapeutic effects 3 days after the first injection when compared to saline treatment. This effect is sustained till 21 days after immunization after which symptoms, as well as body weight loss, start worsening (Fig. 1B, C and E). Interestingly, when TNFR2 is also stimulated before and at first signs of disease, symptoms and body weight loss drastically decrease during the last five days before killing (Fig. 1B, C and E). Simple linear regression analysis testing if time predicted EAE development shows that the overall regression is significant in each treatment group [PBS: *R*^2^ = 0,42, F(1, 271) = 196,7, *p* < 0.0001; EHD2-scTNF_R2_: *R*^2^ = 0,29, F(1, 208) = 87, *p* < 0.0001; ATROSIMAB: *R*^2^ = 0,28, F(1, 250) = 97,9, *p* < 0.0001; E + A:: *R*^2^ = 0,09, F(1, 208) = 21,23, *p* < 0.0001] and that the slopes representing the different treatments greatly differ [F(3, 937) = 19.25, *p* < 0.0001]. Moreover, disease onset is not affected by TNFRs selective targeting (Fig. 1F) while EAE incidence decreases of 10 to 20% in the treatments groups when compared to PBS (Additional file 1: Table S2). This sequential treatment seems to be necessary to exert the protective function of TNFR2 activation.

Furthermore, we asked whether the therapeutic efficacy of ATROSIMAB was affected by anti-drug antibodies (ADA) produced after repeated injections. A precursor molecule of ATROSIMAB (ATROSAB) induced ADA formation in hu/m TNFR1-ki mice abrogating treatment efficacy in two animals [36]. ADA serum level did not correlate with EAE score (Additional file 1: Fig. S3B, C, E and F) or with histopathology (data not shown). We next assessed if the beneficial effects of the sequential treatment with EHD2-scTNF_R2_ and ATROSIMAB was due to decreased level of ADA when compared to single-drug treatments. While the level of ADA against EHD2-scTNF_R2_ is similar in both the EHD2-scTNF_R2_ and the combination group, ATROSIMAB-specific ADA increase when EHD2-scTNF_R2_ is co-administered (Additional file 1: Fig. S3 D). This excludes a detrimental effect of ADA on the therapeutic effects of the sequential treatment described here. Collectively, these data demonstrate an important role of stimulating TNFR2 at early stages of EAE development improving the therapeutical outcome of blocking TNFR1.

### EHD2-scTNF_R2_ and ATROSIMAB do not influence immune cell subsets in spleen and inguinal lymph nodes

**Fig. 2:**
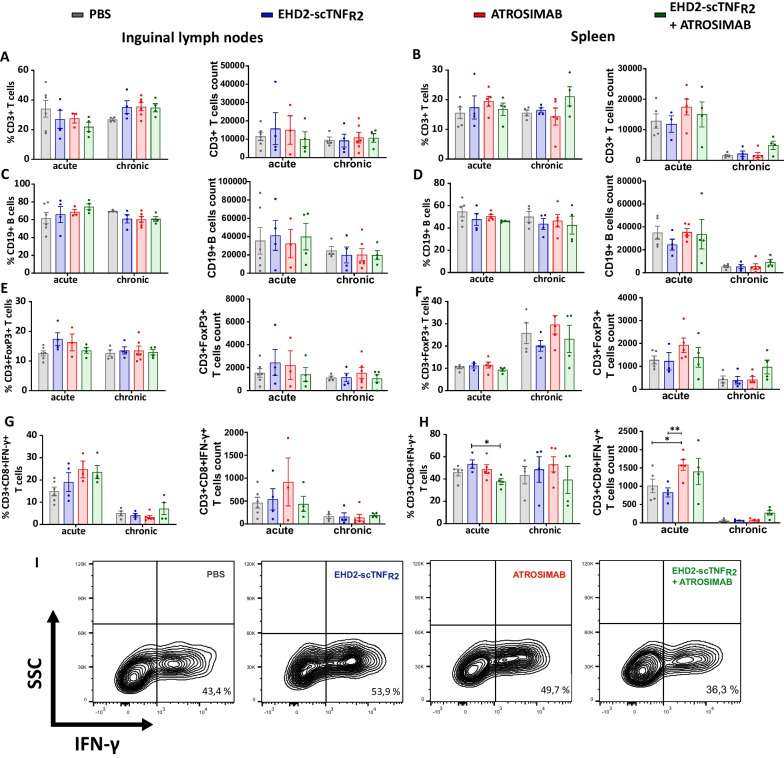
Modulating TNFRs selectively does not lead to major changes in leukocytes subsets. Hu/m TNFR1-ki x hu/m TNFR2-ki mice treated either with saline, EHD2-scTNF_R2_ (TNFR2 agonist), ATROSIMAB (TNFR1 antagonist) or a combination (E + A) were killed either 18 (acute) or 25 (chronic) days post-immunization (dpi; *n* = 3–5 animals/treatment group/killing timepoint). Immune cells from inguinal lymph nodes (on the left) and spleen (on the right) were analyzed by flow cytometry to reveal the frequencies of CD3 + T cells (**A** and **B**), CD19 + B cells (**C** and **D**), CD3 + FoxP3 + Treg (**E** and** F**) and CD3 + CD8 + IFN-γ + cytotoxic T cells (**G** and **H**). Representative contour plots depict interferon gamma (IFN-γ) expression of CD8 + T cells in spleen samples from animals killed at 18 dpi (**I**). The percentage of CD3 + CD8 + IFN-γ + cells is reported at the bottom right of each plot. Data are presented as mean ± SEM and differences between groups were assessed with one-way ANOVA and Tukey’s post-test. *p < 0.05, **p < *0.01*

An abnormal inflammatory response is typical of autoimmune diseases. Specifically, EAE immunopathology comprises activated T helper (Th) cells acquiring pro-inflammatory features by producing IFN-γ and IL-17A, mononuclear immune cell infiltration in the CNS and aberrant innate immune responses [38, 39]. Therefore, we next characterized the immune cell subsets in spleen and inguinal lymph nodes (iLN), draining secondary lymphoid organs greatly affected during EAE. Thirteen different leukocyte subsets were typed, populations including monocytes, neutrophils, B cells, cytotoxic T cells, Treg and Th cells producing IFN-γ, IL-4 or IL-17A (Additional file 1: Fig. S1 and S4). Modulating TNFRs sequentially or individually has no profound impact on these immune cell subsets in spleen and iLN (Additional file 1: Fig. S4). In general, CD3 + T cells and CD19 + B cells are marginally affected by TNFRs selective modulation at both killing timepoints and in both spleen and iLN (Fig. 2A–D). In an autoimmunity context, Treg have the important role of suppressing the activity of pro-inflammatory leukocytes, promoting resolution of inflammation. Even though EHD2-scTNF_R2_ tends to increase the percentage of CD3 + FoxP3 + cells in iLN at 18 dpi, no strong effects on Treg population in the periphery, as previously described [23, 25], were observed (Fig. 2E and F). Interestingly, sequential treatment with EHD2-scTNF_R2_ and ATROSIMAB decreased the number of cytotoxic T cells producing IFN-γ in the spleen at 25 dpi when compared to EHD2-scTNF_R2_ treatment alone but not in the iLN (Fig. 2G and H). Overall, in this model single and sequential treatments do not induce major skewing of immune cell subsets in spleen and iLN organs.

### Sequential modulation of TNFRs decreases demyelination but does not reduce infiltration of T and B cells into the spinal cord

**Fig. 3:**
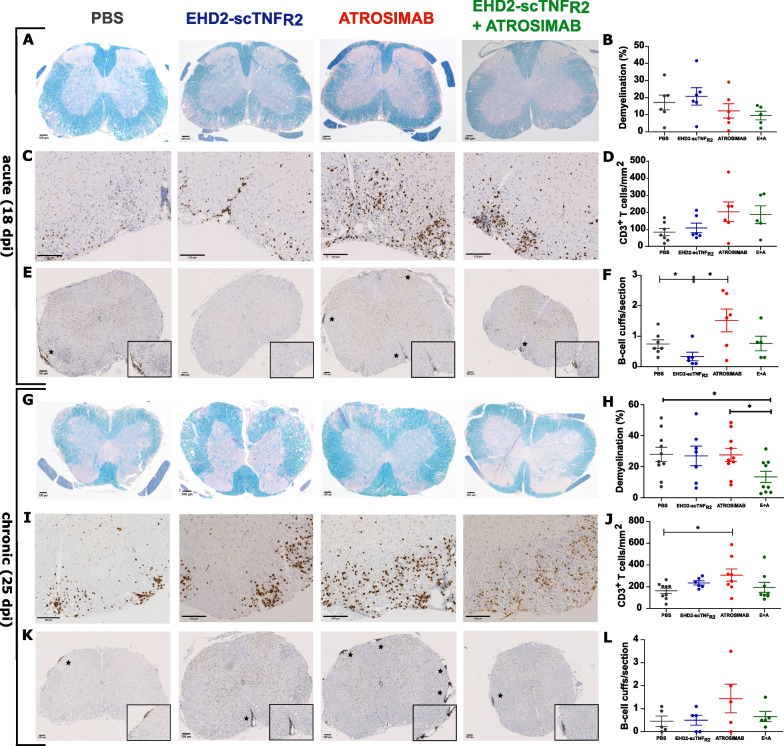
Sequential treatment decreases demyelination while single treatments interfere with lymphocytes recruitment to the CNS. Immunohistochemical analysis of spinal cord sections of hu/m TNFR1-ki x hu/m TNFR2-ki mice immunized with MOG_35-55_ and treated either with saline, EHD2-scTNF_R2_ (TNFR2 agonist), ATROSIMAB (TNFR1 antagonist) or a combination (E + A). Demyelination degree was measured with Klüver Barrera staining at 18 days post-immunization (dpi; *n* = 5–6/group; **A** and **B**) and at 25 dpi (*n* = 7–10/group; **G** and** H**). The number of T cells present in the sections was measured by automated counting of CD3 + cells at 18 dpi (*n* = 6–7/group;** C** and** D**) and 25 dpi (*n* = 6–9/group; **I** and** J**). B-cell clustering was assessed by counting the number of perivascular cuffs (black stars) at 18 dpi (*n* = 5–7/group;** E** and **F**) and 25 dpi (*n* = 5/group; **K** and** L**). Data are presented as mean ± SEM and differences between groups were assessed with Mann–Whitney test. *p < *0.05*

In EAE, the characteristic consequence of autoreactive T cells directed against myelin is lymphocyte infiltration and demyelinating lesions, especially in the SC. Previous research shows decreased demyelination after treatment with TNFR1 antagonists [21, 36], but also with a TNFR2 agonist [23]. Therefore, we analyzed the extent of demyelination in SC sections to determine the impact of manipulating TNF-TNFRs signaling. At the acute stage of EAE, two injections of ATROSIMAB with or without EHD2-scTNF_R2_ reduce demyelination, but this difference did not reach significance (Fig. 3A and B). In contrast with previous studies, we show that mice receiving treatment with a single compound develop demyelination in the SC similarly to saline-treated mice (Fig. 3G and H). Modulating both TNFRs is required to significantly decrease demyelination at 25 dpi (Fig. 3G and H). Comparison of demyelination and clinical scores confirms that the stronger the treatment effect on paralysis symptoms, the lower the demyelination (Figs. 1A, 3B and H).

Additionally, we stained for Alzheimer precursor protein (APP) in SC sections from a small cohort of mice and compared it with demyelination. We found APP^+^ axonal spheroids spatially associated with demyelinated sites and their number matches the amount of demyelination (Additional file 1: Fig. S5).

Lymphocyte infiltration into CNS tissue from peripheral circulation is another pathological consequence of MOG_35-55_ immunization [40]. Therefore, we measured the number of T and B cells present in SC sections and observed no effects of sequential TNFRs modulation or EHD2-scTNF_R2_ treatment compared to saline treatment. Interestingly, two injections of ATROSIMAB increase T cell count in the SC at 18 dpi (Fig. 3C and D), an effect that becomes significant after three injections (Fig. 3I and J). Furthermore, here single ATROSIMAB treatment also increases the number of CD19+ B cells clustered at perivascular sites at 18 dpi but this effect reaches significance only when compared to EHD2-scTNF_R2_ treatment at 18 dpi (Fig. 3E and F). Interestingly, TNFR2 agonism instead decreases the number of meningeal B-cell cuffs present at 18 dpi (Fig. 3E and F), an effect that does not occur upon one injection of ATROSIMAB following EHD2-scTNF_R2_ treatment. At the chronic disease stage, the disease features overall are similar, except for EHD2-scTNF_R2_ treatment that induces B-cell cuffing similarly to PBS (Fig. 3K and L). Altogether, both single treatments affect the number of infiltrated T and B cells while having mild effects in decreasing demyelination. Modulating both receptors sequentially dampens demyelination independently from B- to T-cells infiltration in the CNS.

### Regulatory T cells increase in number in the spinal cord upon TNFR2 stimulation

**Fig. 4:**
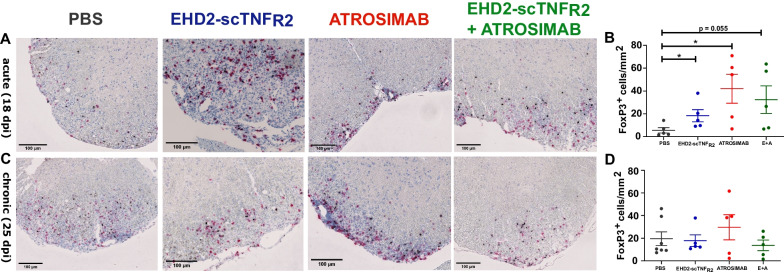
TNFR2 stimulation leads to an increased number of Tregs in the spinal cord at 18 dpi. Immunohistochemical analysis of FoxP3 staining of hu/m TNFR1-ki x hu/m TNFR2-ki mice immunized with MOG_35-55_ and treated either with saline, EHD2-scTNF_R2_ (TNFR2 agonist), ATROSIMAB (TNFR1 antagonist) or a combination (E + A). FoxP3 + cells (black) were counted in spinal cord sections of mice killed at 18 days post-immunization (dpi; *n* = 5/group; **A** and **B**) and at 25 dpi (*n* = 5–7/group; **C** and **D**). Data are presented as mean ± SEM and differences between groups were assessed with Mann–Whitney test. *p < *0.05*

Furthermore, we asked whether Treg migrated from the circulation into the CNS tissue. Immunohistochemical analysis of FoxP3 + cells revealed a fourfold increase of Treg infiltration into the SC parenchyma upon TNFR2 stimulation when compared to saline control (Fig. 4A and B). This effect is robust at the acute stage of disease, but disappears at the chronic stage (Fig. 4C and D). Control mice killed at 25 dpi show similar number of Treg as EHD2-scTNF_R2_-treated mice killed at 18 dpi (Fig. 4B and D). This effect highlights that TNFR2 stimulation accelerates Treg presence in SC. In general, mice receiving ATROSIMAB treatment display a high degree of variation in the number of Treg in the SC (Fig. 4B and D). Nevertheless, ATROSIMAB effect on Treg presence in the SC is significant when compared to PBS at acute stage of disease (Fig. 4A and B). Therefore, blocking TNFR1 lacks consistent, yet relevant, effects on Treg expansion and migration into the CNS. Taken together, these results highlight an important role of TNFR2 stimulation in Treg accumulation into the CNS, whereas peripheral immune cell subsets remain mostly unchanged after TNFRs modulation in EAE.

## Discussion

Here, we demonstrate that activating TNFR2 and blocking TNFR1 persistently decrease paralysis symptoms and body weight loss in EAE mice. Even though blocking of TNFR1 shows therapeutic effects, paralysis symptoms and body weight loss start worsening a few days after the last treatment. To achieve prolonged and robust therapeutic effects on motor disease and demyelination, TNFR1 antagonism requires additional TNFR2 stimulation. While single treatments differentially affect lymphocyte recruitment to the CNS, the sequential treatment does not interfere with T- and B-cell presence in the spinal cord. When analyzing treatment effects in secondary lymphoid organs such as spleen and iLN, single and sequential treatments did not induce major shift in the frequencies of various immune cell subsets.

In our study, single EHD2-scTNF_R2_ treatment starting three days before the onset of motor symptoms does not lead to a substantial decrease in EAE symptoms and pathology. EHD2-scTNF_R2_ tends to increase CD3 + FoxP3 + Treg in iLN but not significantly and accelerates their infiltration into the spinal cord. On the other hand, TNFR2 stimulation induces Treg expansion in different CNS and non-CNS disease models [24, 41–44], promoting its immune regulating functions during pathological autoimmunity [25, 45–48]. Of interest, Treg distributed in CNS push oligodendrocyte’s progenitor cells (OPCs) differentiation and remyelination in vitro and in vivo [49]. Thus, it may be that a more robust and profound effect on the expansion of peripheral Treg is needed to observe considerable effects on motor symptoms and demyelination. It should be further investigated whether TNFR2-stimulated Treg start remyelination at early stages of disease and if simultaneous blocking of TNFR1 is critical to prevent ongoing demyelination, allowing Treg to exert their remyelinating function.

We show that injections of a mouse TNFR1 antagonistic antibody at 9 and 13 dpi significantly decreases EAE symptoms and demyelination when hu/m TNFR2-ki are killed at 18 dpi (Additional file 1: Fig. S2A, B and F). On the other hand, ATROSIMAB protects mice from developing severe symptoms at the acute phase of EAE (18 dpi) but loses its effect during later stage of disease (22–25 dpi). Further, ATROSIMAB injections at 12 and 15 dpi do not exert the same effect on demyelination (Fig. 3A and B) as in the combination study (Additional file 1: Fig. S2E and F). Recently, ATROSIMAB treatment was shown to be beneficial in different inflammation models such as experimental arthritis, non-alcoholic steatohepatitis (NASH) and also in EAE [21]. Previously, four injections with a monoclonal antibody blocking TNFR1 after EAE onset led to a decrease of symptoms and SC pathology. After 35 days, animals experience disease relapse and re-treatment further prolonged the beneficial effects of blocking TNFR1 [36]. It is conceivable that repeating treatment four times and increasing the dose to 30 mg/kg are necessary in order to achieve stronger impact of ATROSIMAB treatment on EAE symptoms and pathology at chronic EAE stage.

The intricate nature of TNF-TNFRs signaling and expression by both leukocytes and by CNS cell types poses some challenges in analyzing therapeutical effects in autoimmune diseases. Our findings revealed that blocking TNFR1 promotes B-cell migration to the CNS and clustering between endothelial cells and the basal membrane (Robin–Virchow Space), reflecting meningeal ectopic B-cell follicles in MS [50]. This meningeal inflammation was previously shown to be triggered by astrocyte-specific tmTNF signaling through TNFR2 in transgenic mice not expressing TNFR1 [29]. Additionally, this study shows that TNF-induced oligodendrocytes apoptosis and primary demyelination are specifically mediated by TNFR1. Collectively, imbalance in TNF-TNFRs signaling seems to affect lymphocyte recruitment to the CNS and development of neuropathology. Our data show that sequential modulation of TNFRs aimed at rebalancing TNF-TNFRs signaling decreases demyelination and does not influence T- and B-cell presence in both SC and periphery.

Furthermore, we found that most of the B cells, if not all, clustered in the perivascular space do not express immunoglobulin (Additional file 1: Fig. S2L). Possibly, blocking TNFR1 likely enhances tmTNF-TNFR2 signaling that could in turn promote the differentiation of B cells into a more regulatory phenotype. Nevertheless, direct stimulation of TNFR2 with EHD2-scTNF_R2_ decreases cuffing of B cells at perivascular sites and does not alter CD19 + B-cell count in secondary lymphoid organs. So far, several studies highlighted an anti-inflammatory and protective function of regulatory T and B cells in autoimmunity. For instance, B cells activated through toll-like receptor 9 (TLR9) express TNFR2 and differentiate into IL-10-producing regulatory B cells (Breg) [51]. Furthermore, RA patients treated with the TNF inhibitor adalimumab show increased expression of tmTNF on monocytes which bind to TNFR2 expressed by Treg. This leads to Treg expansion and their subsequent suppression of Th17-driven pro-inflammatory responses [46]. Another interesting finding is that adoptive transfer of Breg in EAE mice promotes expansion of Treg, thereby suppressing autoimmunity [52]. Therefore, future research should focus on characterizing lymphocytes’ phenotype upon treatment with TNFRs modulators, their distributions in relevant tissues and their impact on EAE and MS pathology.

## Conclusions

Collectively, our results suggest an aiding role of stimulating TNFR2 followed by blocking TNFR1 against EAE paralysis and demyelination. This therapeutic effect is superior to single-drug treatments and may have an important role in redirecting immune cells to regulatory function and their migration into the CNS. Given the profound effects of manipulating TNF-TNFRs signaling in an autoimmunity context reported here and in literature, we consider this pharmacological approach to have high relevance for future research into MS treatment. The results of this sequential treatment approach demonstrated in the EAE model for MS offer a new perspective on the potential benefits of targeting TNFRs selectively at different stages of disease development to improve overall treatment efficacy. Further studies in appropriate clinical settings are needed to determine the actual impact and feasibility of this treatment approach.

## Supplementary Information

**Additional file 1. Figure S1.** Example of the gating strategy used to distinguish different immune cell subsets. Representative spleen sample from a PBS-treated hu/m TNFR2-ki mouse sacrificed at 25 days post immunization. **Figure S2.** Blocking TNFR1 with and without a TNFR2 agonist limits EAE development by decreasing demyelination while increasing lymphocyte numbers in the spinal cord. **Figure S3.** EHD2-scTNF_R2_ and ATROSIMAB treatments increase the level of anti-drug antibodies in serum but do not affect clinical EAE development. **Figure S4.** Frequency of immune cell subsets in inguinal lymph nodes and spleen. **Figure S5.** APP staining of spinal cord sections from representative mice from the acute cohort shows that axonal degeneration increases similarly to demyelination. **Table S1.** Primary antibodies used for immunohistochemistry and flow cytometry. **Table S2.** Clinical EAE development in hu/m TNFR1-ki x hu/m TNFR2 -ki mice treated either with PBS, a human TNFR2 agonist (EHD2-scTNF_R2_) and/or a human TNFR1 antagonist (ATROSIMAB).

## Data Availability

All data generated or analyzed during this study are included in this published article [and its additional information files].

## Abbreviations

ADA: Anti-drug antibody
AD: Alzheimer’s disease
APP: Alzheimer precursor protein
AS: Ankylosing spondylitis
AUC: Area under the curve
Bregs: Regulatory B cells
CaI: Ionomycin
CD: Crohn’s disease
CNS: Central nervous system
DAB: 3,3′ Diaminobenzidine-tetra-hydrochloride
Dpi: Days post-immunization
EAE: Experimental autoimmune encephalomyelitis
FCS: Fetal calf serum
hu/m TNFR-ki: Human/murine TNFR knock-in
iLN: Inguinal lymph node
i.p.: Intraperitoneal
Ig: Immunoglobulin
KO: Knock-out
LFB: Luxol Fast Blue
MS: Multiple sclerosis
NASH: Non-alcoholic steatohepatitis
OPCs: Oligodendrocytes’ progenitor cells
PFA: Paraformaldehyde
PS: Penicillin–streptomycin
PTX: Pertussis toxin
P/W: Permeabilization/wash™ buffer
RA: Rheumatoid arthritis
SC: Spinal cord
s.c.: Subcutaneous
SEM: Standard error of the mean
sTNF: Soluble tumor necrosis factor-alpha
Th: T helper
TLR9: Toll-like receptor 9
tmTNF: Transmembrane tumor necrosis factor-alpha
TNF: Tumor necrosis factor-alpha
TNFR: Tumor necrosis factor-alpha receptor
Treg: Regulatory T cell
UC: Ulcerative colitis
WT: Wild type
