# Supplementary material for: Sequential treatment with a TNFR2 agonist and a TNFR1 antagonist improves outcomes in a humanized mouse model for MS
Source: J Neuroinflammation. 2023 May 3;20:106. doi: 10.1186/s12974-023-02785-y (PMC10157968; doi:10.1186/s12974-023-02785-y)
Supplement: Supplementary file 1 — Additional file 1. Figure S1. Example of the gating strategy used to distinguish different immune cell subsets. Representative spleen sample from a PBS-treated hu/m TNFR2-ki mouse sacrificed at 25 days post immunization. Figure S2. Blocking TNFR1 with and without a TNFR2 agonist limits EAE development by decreasing demyelination while increasing lymphocyte numbers in the spinal cord. Figure S3. EHD2-scTNFR2 and ATROSIMAB treatments increase the level of anti-drug antibodies in serum but do not affect clinical EAE development. Figure S4. Frequency of immune cell subsets in inguinal lymph nodes and spleen. Figure S5. APP staining of spinal cord sections from representative mice from the acute cohort shows that axonal degeneration increases similarly to demyelination. Table S1. Primary antibodies used for immunohistochemistry and flow cytometry. Table S2. Clinical EAE development in hu/m TNFR1-ki x hu/m TNFR2 -ki mice treated either with PBS, a human TNFR2 agonist (EHD2-scTNFR2) and/or a human TNFR1 antagonist (ATROSIMAB). [file 12974_2023_2785_MOESM1_ESM.pdf]

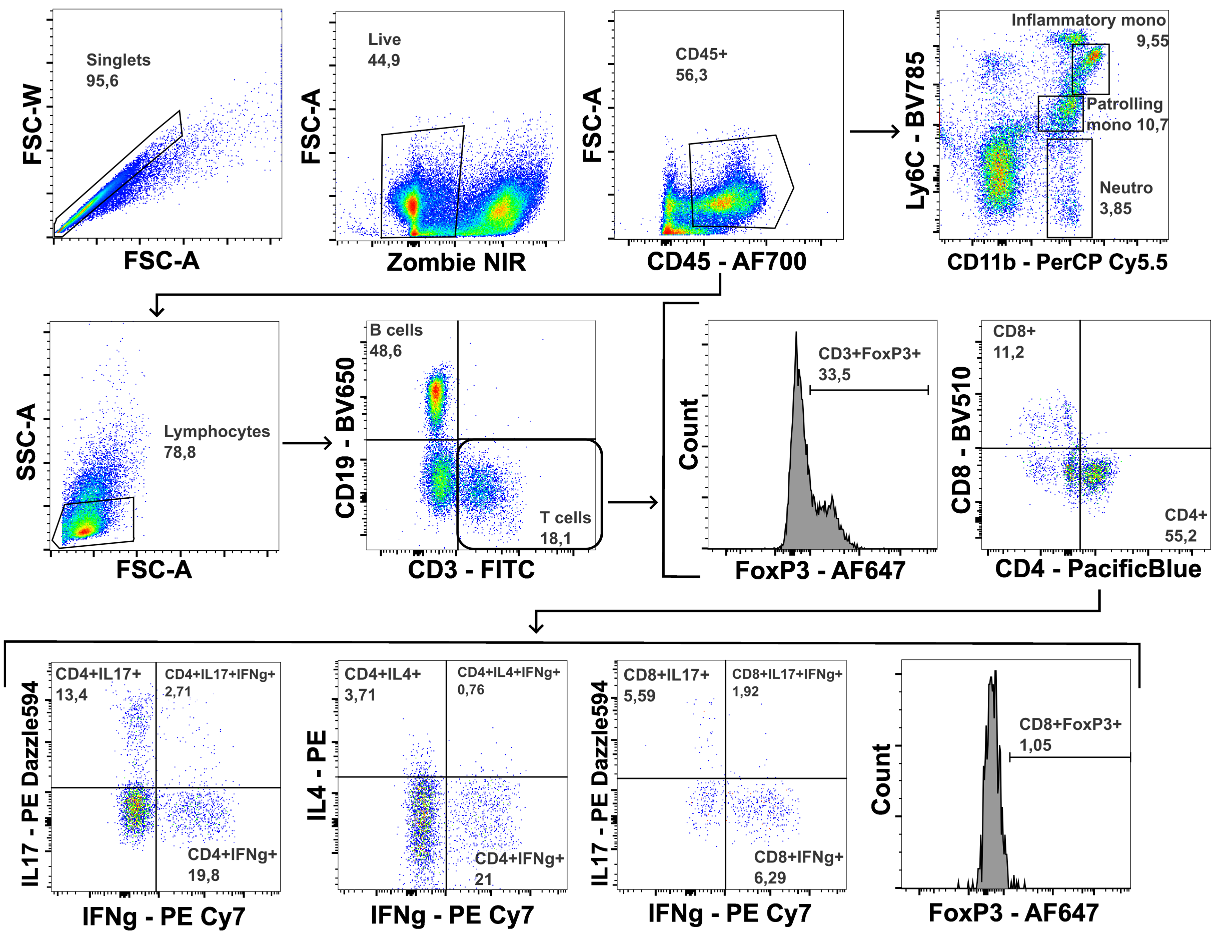


*Fig. S1. Example of the gating strategy used to distinguish different immune cell subsets. Representative spleen sample from a PBS-treated hu/m TNFR2-ki mouse sacrificed at 25 days post immunization.*

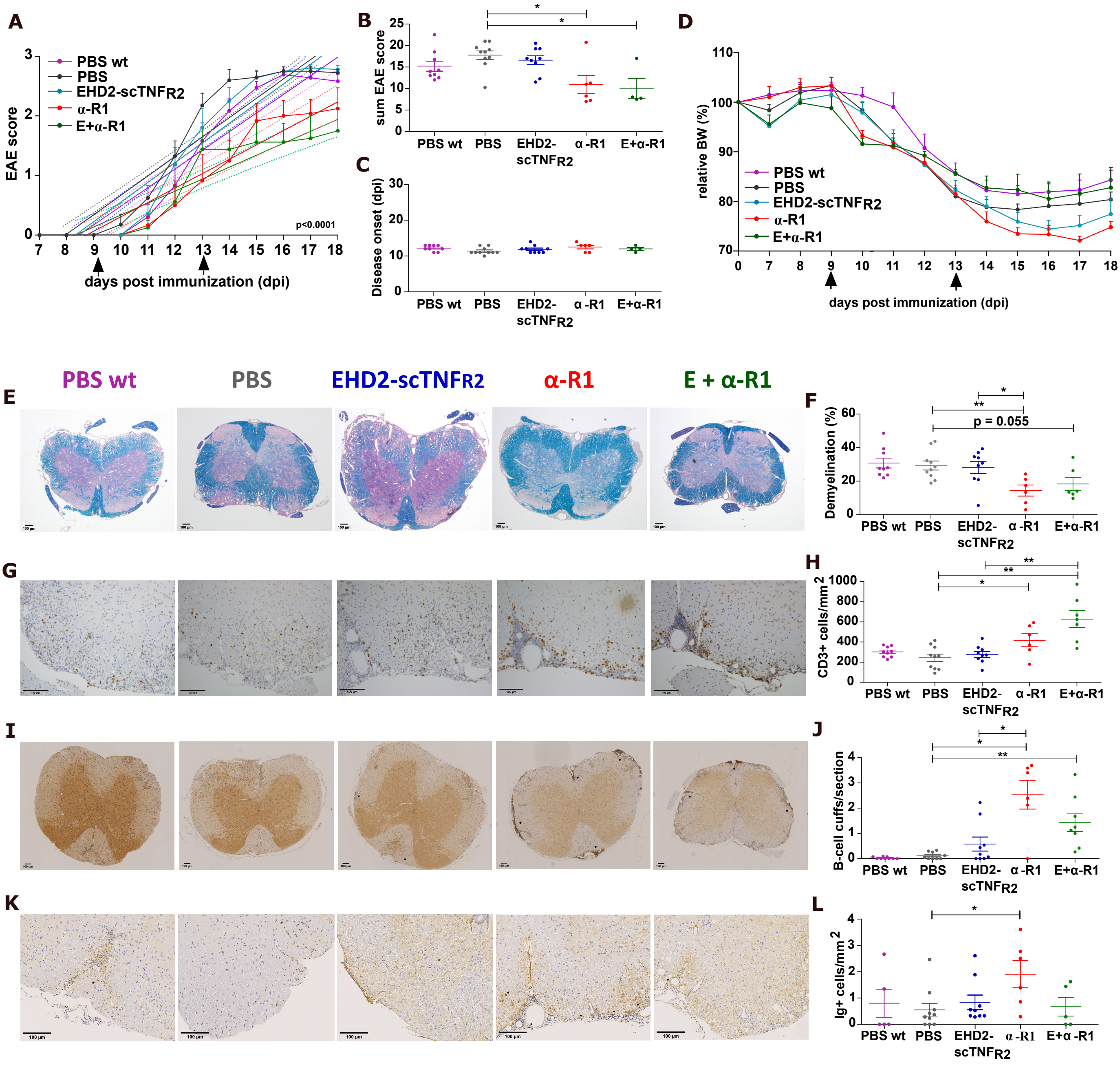


*Fig. S2. Blocking TNFR1 with and without a TNFR2 agonist limits EAE development by decreasing demyelination while increasing lymphocyte numbers in the spinal cord*. Hu/m TNFR2-ki and WT mice were immunized with $MOG_{35\text{-}55}$ and treated at 9- and 13-days post immunization (dpi; **black arrows**) either with saline (n= 10/genotype), EHD2-scTNF*R2* (TNFR2 agonist; n= 9; 10 mg/kg), anti-mouse TNFR1 (α-R1; n= 6; 20 mg/kg) or a combination (E+ α-R1; n=4). Disease development (**A**, **B** and **C**) and body weight (BW; **D**) were measured daily from 7 to 18 dpi. Mice were sacrificed at 18 dpi and spinal cord sections were analyzed as follows. Demyelination degree was measured with Klüver Barrera staining (n= 6-10/group; **E** and **F**). The number of T cells present in the sections was measured by counting CD3 positive cells per $mm^2$ (n= 6-10/group; **G** and **H**). B-cell clustering was assessed by counting the number of B220 positive cuffs (**black stars**) present in the perivascular space (n= 6-9/group; **I** and **J**) while Ig staining revealed the number of plasma cells per $mm^2$ (**black stars**, n= 5-10/group; **K** and **L**). Data are presented as mean ± SEM and differences between groups were assessed with Mann-Whitney test, except for body weight loss for which two-way ANOVA and Tukey's post-test were used. **p<0.05, **p<0.01*

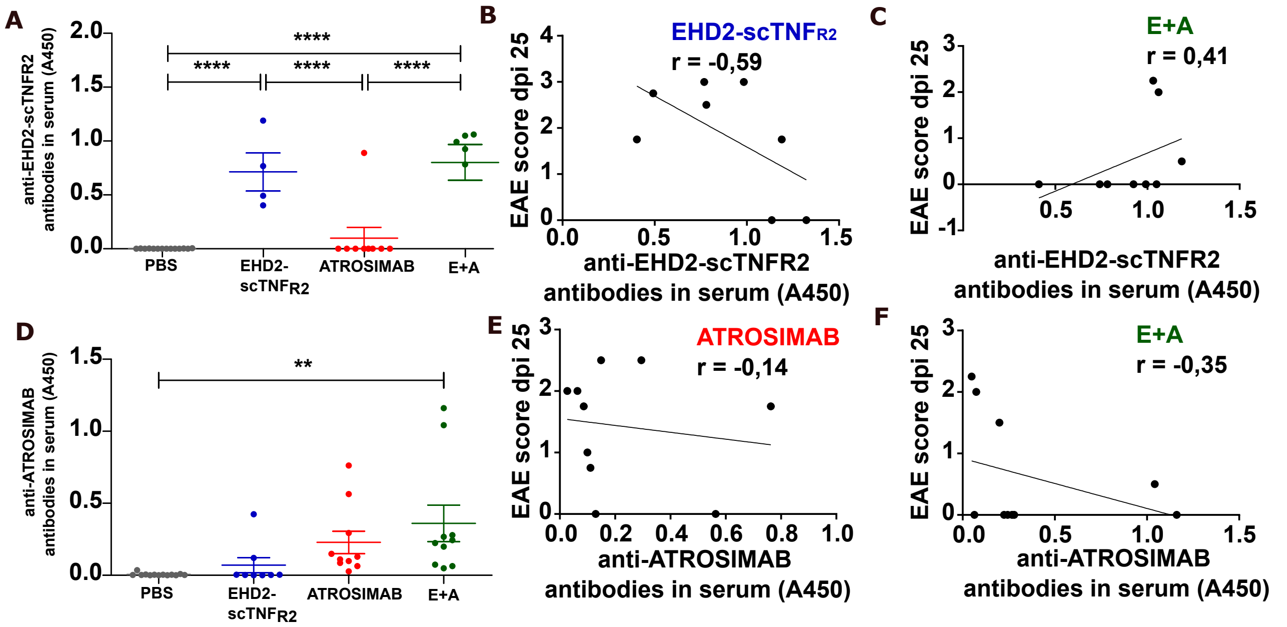


***Fig. S3.*** *EHD2-scTNFR2 and ATROSIMAB treatments increase the level of anti-drug antibodies in serum but do not affect clinical EAE development. Levels of EHD2-scTNFR2-specific antibodies (A) and ATROSIMAB-specific antibodies (D) in serum of hu/m TNFR1-ki x hu/m TNFR2-ki were measured with ELISA and correlated (Pearson coefficient r) with EAE score of animals treated with EHD2-scTNFR2 (TNFR2 agonist; B), ATROSIMAB (TNFR1 antagonist; E) and combination (E+A; C and F) at time of sampling (25 days post immunization, dpi). *p<0.05, **p<0.01, ***p<0.001, ****p<0.0001*

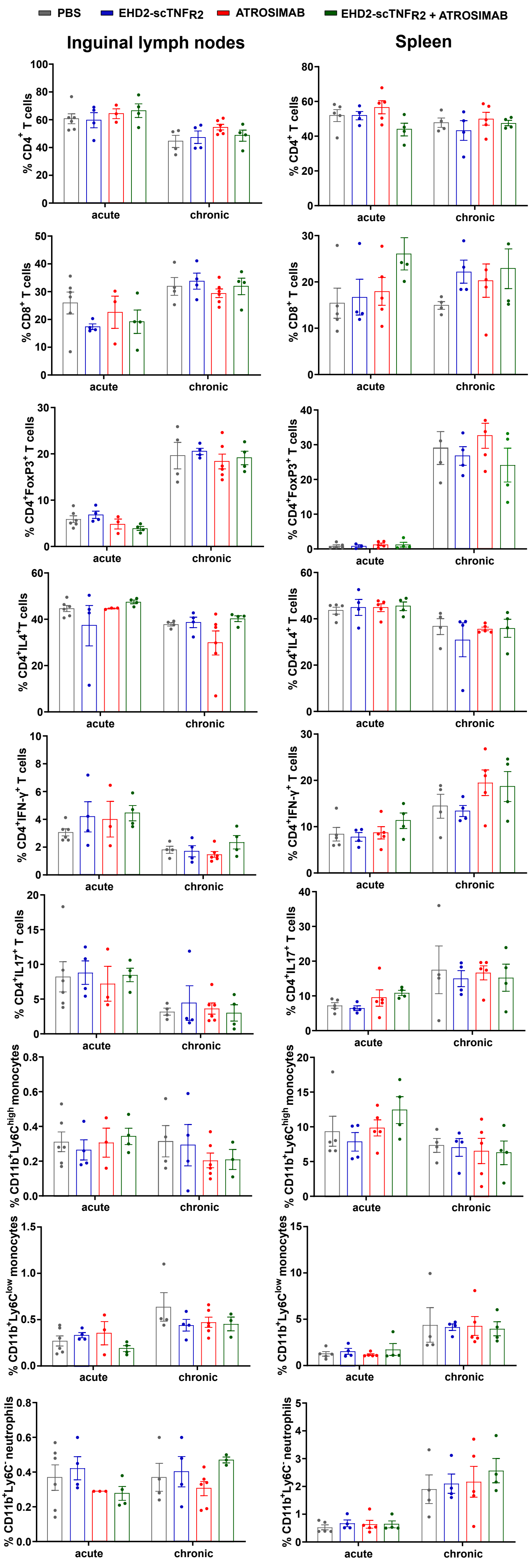


**Fig. S4.** *Frequency of immune cell subsets in inguinal lymph nodes and spleen.*

Hu/m TNFR1-ki x hu/m TNFR2-ki mice were immunized with $MOG_{35-55}$ and treated either with PBS, EHD2-scTNFR2 (TNFR2 agonist), ATROSIMAB (TNFR1 antagonist) or a combination. Immune cell subsets from spleen and inguinal lymph nodes of mice sacrificed either 18- (acute) or 25-days (chronic) post immunization were analyzed by flow cytometry using the gating strategy described in Figure S3.

CD = cluster of differentiation; IFN-γ = interferon gamma; IL = interleukin.

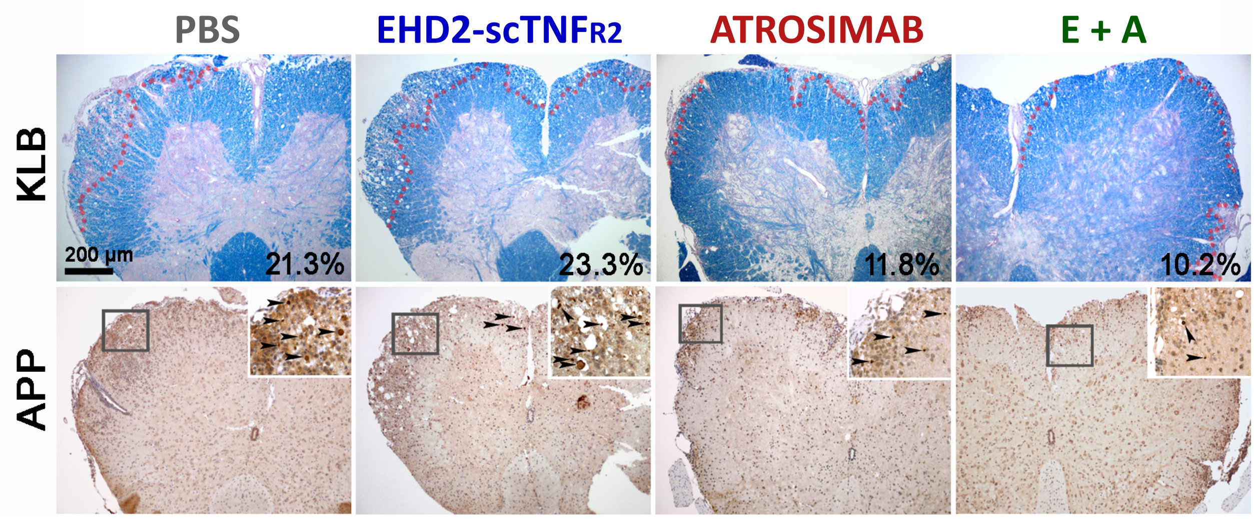


***Figure S5****. APP staining of spinal cord sections from representative mice from the acute cohort shows that axonal degeneration increases similarly to demyelination.* Immunohistochemical analysis of Alzheimer precursor protein (APP) staining of hu/m TNFR1-ki x hu/m TNFR2-ki mice immunized with $MOG_{35-55}$ and treated either with saline, EHD2-$scTNF_{R2}$ (TNFR2 agonist), ATROSIMAB (TNFR1 antagonist) or a combination (E+A). Within the same animal, the number of APP+ cells (black arrowheads) were compared with the degree of demyelination (Kluver-Barrera-KLB). The percentage of demyelination of each animal is depicted at the bottom right of each picture. Only spinal cord sections from animals sacrificed 18 days after immunization (acute cohort) were used for this staining. APP antibody (clone 22C11, Millipore, 1:1000). Scale bar is 200 µm.

***Table S1***. *Primary antibodies used for immunohistochemistry and flow cytometry*

| **Primary antibody** | **Clone** | **Company** | **Target** | **Assay** |
|---|---|---|---|---|
| Alzheimer Precursor Protein (APP) | 22C11 | Millipore | Neurons | Immunohistochemistry |
| CD3 | SP7 | Neomarkers | T cells | Immunohistochemistry |
| FoxP3 | PCH101 | eBioscience | Regulatory T cells | Immunohistochemistry |
| CD19 | D4V4B | Cell Signaling | B cells | Immunohistochemistry |
| CD45R/B220 | RA3-6B2 (RUO) | BD Pharmingen | B cells | Immunohistochemistry |
| IgG | - | Jackson | Plasma cells | Immunohistochemistry |
| CD3-FITC - extracellular | 17A2 | Biolegend | T cells | Flow cytometry |
| CD4-PB - extracellular | GK1.5 | Biolegend | T helper cells | Flow cytometry |
| CD45-AF700 - extracellular | 30-F11 | Biolegend | Leukocytes | Flow cytometry |
| CD8-BV510 - extracellular | 53-6.7 | Biolegend | Cytotoxic T cells | Flow cytometry |
| CD19-BV650 - extracellular | 6D5 | Biolegend | B cells | Flow cytometry |
| CD11b-PerCP/Cy5.5 - extracellular | M1/70 | Biolegend | Myeloid cells | Flow cytometry |
| Ly6C-BV785 - extracellular | HK1.4 | Biolegend | Myeloid cells | Flow cytometry |
| IL-4-PE - intracellular | 11B11 | Biolegend | IL-4 secreting cells | Flow cytometry |
| IL-17-PE/Dazzle594 - intracellular | TC11-18H10.1 | Biolegend | IL-17 secreting cells | Flow cytometry |
| IFN-γ-PE/Cy7 - intracellular | XMG1.2 | Biolegend | IFN-γ secreting cells | Flow cytometry |
| Foxp3-AF647 - intracellular | MF-14 | Biolegend | Regulatory T cells | Flow cytometry |

***Table S2.*** *Clinical EAE development in hu/m TNFR1-ki x hu/m TNFR2 -ki mice treated either with PBS, a human TNFR2 agonist (EHD2-scTNF$_{R2}$) and/or a human TNFR1 antagonist (ATROSIMAB).*

| Treatment group | Day of disease onset | Sum EAE score | AUC EAE score | Mean EAE score | Median EAE score | Max EAE score | Days with EAE score ≥ 2 | EAE disease incidence | Death incidence due to EAE |
|---|---|---|---|---|---|---|---|---|---|
| **PBS** | 12.8 ± 0.7 | 26.9 ± 3.7 | 25.9 ± 3.6 | 1 ± 0.1 | 0.8 ± 0.2 | 2.5 ± 0.2 | 9.5 ± 1.4 | 13/13 (100%) | 1/13 (7.7%) |
| **EHD2-scTNF$_{R2}$** | 13.5 ± 0.9 | 25.3 ± 3.0 | 24.3 ± 2.9 | 0.9 ± 0.1 | 0.3 ± 0.2 | 2.6 ± 0.1 | 7.5 ± 1.6 | 8/10 (80%) | 1/10 (10%) |
| **ATROSIMAB** | 13.9 ± 0.9 | 15.9 ± 3.3 | 15.1 ± 3.2 | 0.6 ± 0.1 | 0.5 ± 0.2 | 2 ± 0.2 *[a] | 3.2 ± 1.3 *[a] | 11/12 (91.7%) | 0/12 (0%) |
| **EHD2-scTNF$_{R2}$ + ATROSIMAB** | 13.6 ± 1.4 | 12.2 ± 3.3 *[a], *[b] | 11.9 ± 3.2 *[a], *[b] | 0.4 ± 0.1 *[a], *[b] | 0.2 ± 0.1 *[a] | 1.6 ± 0.2 **[a], *[b] | 2.3 ± 1.2 **[a], *[b] | 9/10 (90%) | - 0/10 (0%) |

Data are presented as mean ± SEM and difference s between groups were assessed with Mann-Whitney test, except for disease onset and days with EAE score ≥ 2 for which one way ANOVA and Tukey’s post test were used. a = compared to PBS and b = compared to EHD2-scTNFR2. *p<0.05, **p<0.01
